# Primary Congenital Hypothyroidism in Children Below 3 Years Old - Etiology and Treatment With Overtreatment and Undertreatment Risks, a 5-Year Single Centre Experience

**DOI:** 10.3389/fendo.2022.895507

**Published:** 2022-06-27

**Authors:** Elżbieta Lipska, Agnieszka Lecka-Ambroziak, Daniel Witkowski, Katarzyna Szamotulska, Ewa Mierzejewska, Mariusz Ołtarzewski

**Affiliations:** ^1^ Endocrinology Outpatient Clinic, Institute of Mother and Child, Warsaw, Poland; ^2^ Department of Endocrinology and Diabetology, Children’s Memorial Health Institute, Warsaw, Poland; ^3^ Department of Epidemiology and Biostatistics, Institute of Mother and Child, Warsaw, Poland; ^4^ Department of Screening and Metabolic Diagnostics, Institute of Mother and Child, Warsaw, Poland

**Keywords:** congenital hypothyroidism, permanent CH, transient CH, neurodevelopment, neonatal screening, TSH threshold, CH overtreatment, CH undertreatment

## Abstract

Worldwide neonatal screening for congenital hypothyroidism (CH) is a gold standard of active surveillance in newborns. Prompt diagnosis, subsequent timely treatment implementation, and proper dosage of levothyroxine (L-T4) are crucial for normal growth and development, especially of the central nervous system. However, overtreatment may have a potential negative impact on further neurodevelopment. We retrospectively analysed data of 99 newborns with CH diagnosis, referred to the Endocrinology Outpatient Clinic of the Institute of Mother and Child in Warsaw, Poland from the CH screening program from 2017 to 2021. We evaluated the diagnostic process and treatment up to the age of 3 years. We compared groups of children from the first and the second screening groups (FSG, SSG) in the neonatal screening with an evaluation of ultrasound examination (thyroid dysgenesis vs. gland *in situ*, GIS). The overtreatment and undertreatment risks were assessed and an analysis of the new TSH thresholds was performed. Treatment was implemented at a median of 9 days of life (3 – 27); 8 days (3 – 17) in FSG and 19 (6 – 27) in SSG. The dose of L-T4 differed between FSG and SSG at all three analysed time points (start of the therapy, 12 months, and 3 years) with significantly higher doses in FSG. The same was observed for the patients with thyroid dysgenesis vs. GIS. Screening TSH level was ≥ 28mIU/l in 91.7% of patients with thyroid dysgenesis in comparison to 74.0% of patients with GIS (p= 0.038). The optimally treated group (fT4 in the upper half of the reference range, according to the guidelines) was up to 58.0% of the children during the follow-up. The risk for overtreatment was present in 1/5 of the study group after 12 months and 1/4 after 3 years of L-T4 therapy. Analysis of new TSH thresholds showed an increased prevalence of mild hypothyroidism, GIS, and either euthyroid state or overtreatment while treating with lower L-T4 doses in comparison to the rest of the cohort. The study confirmed the general efficacy of the CH diagnostic pathway and the timely implemented L-T4 therapy. The suspected overtreatment after the first 12 months of L-T4 therapy requires consideration of the earlier diagnosis re-evaluation.

## Introduction

The thyroid hormones (THs) are essential for a harmonious development process in early life. Congenital hypothyroidism (CH) is the most prevalent endocrine disorder in newborns, infants, and children in early childhood, especially below 3 years of age. TH deficiency begins in foetal life due to thyroid axis disturbances. CH may be primary (thyroid gland disorders) or secondary/tertiary (pituitary/hypothalamic disorders); each of them can be temporary (TCH) or permanent (PCH). The incidence of primary CH in the iodine-sufficient areas, which is the topic of this paper, is estimated as 1 in 2000 up to 1 in 3000 live births (1). Currently, we observe an increasing incidence of primary CH, which can be related to the detection of milder cases of CH, and increased survival of preterm neonates (2). In the first years of the screening, the incidence was reported as 1:3000 – 1:4000, but in recent years, it has also been documented as 1:1400 - 1:2800 (3–5).

It is well known that THs play a fundamental role in tissue and organ development in early life. Their role begins in early embryonic life, when in the first half of pregnancy the foetus needs a transplacental transfer of maternal THs. Subsequently the foetal TH production progresses (6). THs optimize critical processes of neurodevelopment and brain maturation, growth and mental development, as well as metabolism (7). In the central nervous system (CNS) the main effects of TH deficiency include deficits in neuronal migration and proliferation, decreased expression of neuronal differentiation factors, reduced cortical thickness, cortical dysplasia, impaired dendrites and axons development, decreased expression of proteins involved in synaptic plasticity, and delayed myelinization (7) and hippocampal development, which is essential for learning and memory functions (6). These complex influences may result in permanent CNS disorders, cause neurological and psychiatric deficits, intellectual disability, spasticity, disturbances of gait and coordination (6), and hearing/attention deficits (8), which cannot be completely resolved with delayed treatment implementation (9).

During the adaptation period in newborns, the THs are also essential for thermogenesis and energy production. THs also influence the cardiovascular system and TH deficiency may cause impaired cardiac diastolic function, increased intima-media thickness, and reduction of exercise capacity and cardiopulmonary function in adult life, despite further proper replacement therapy (10).

The main CH cause worldwide, especially in Africa and Asia, is still iodine deficiency, leading to goitre, deleterious forms of intellectual disability, and in the foetal life – stillbirths or spontaneous abortions, or congenital anomalies (cretinism) (11). However, in the European countries and others with sufficient iodine supply, e.g., in North America, other reasons predominate, among them thyroid dysgenesis (>50%), including athyreosis in 20%-30%, increasing incidence of thyroid dyshormonogenesis (30%-46%), and secondary CH (≈5%) (6, 12–14). In the Polish general population, the adequate iodine intake has been provided due to mandatory iodisation of household salt since 1997 (15).

Thyroid dysgenesis includes agenesis, hypotrophy, hemiagenesis, and ectopy of the thyroid gland. In some cases, especially with family history of CH, gene mutations can be confirmed, including *NKX2-1, NKX2-5, FOXE1, PAX8, TSHR, GNAS, GLIS3, CDCA8, JAG1* genes (2, 6). Thyroid dyshormonogenesis usually is caused by single gene mutations, encoding globulins involved in TH synthesis (thyroid peroxidase - *TPO*, thyroglobulin - *TG*, Na+/I- symporter – *SCL5A5/NIS*, pendrine – *SCL26A4/PDS*), or directly influencing TH synthesis and metabolism (*DUOX2, DUOX2A, IYD/DEHAL1*) *(* 6). The secondary/tertiary CH is the rarest, with an incidence of 1:16,000-50,000, and includes pituitary and/or hypothalamus defects as well as TH resistance (1, 16).

**Figure 1:**
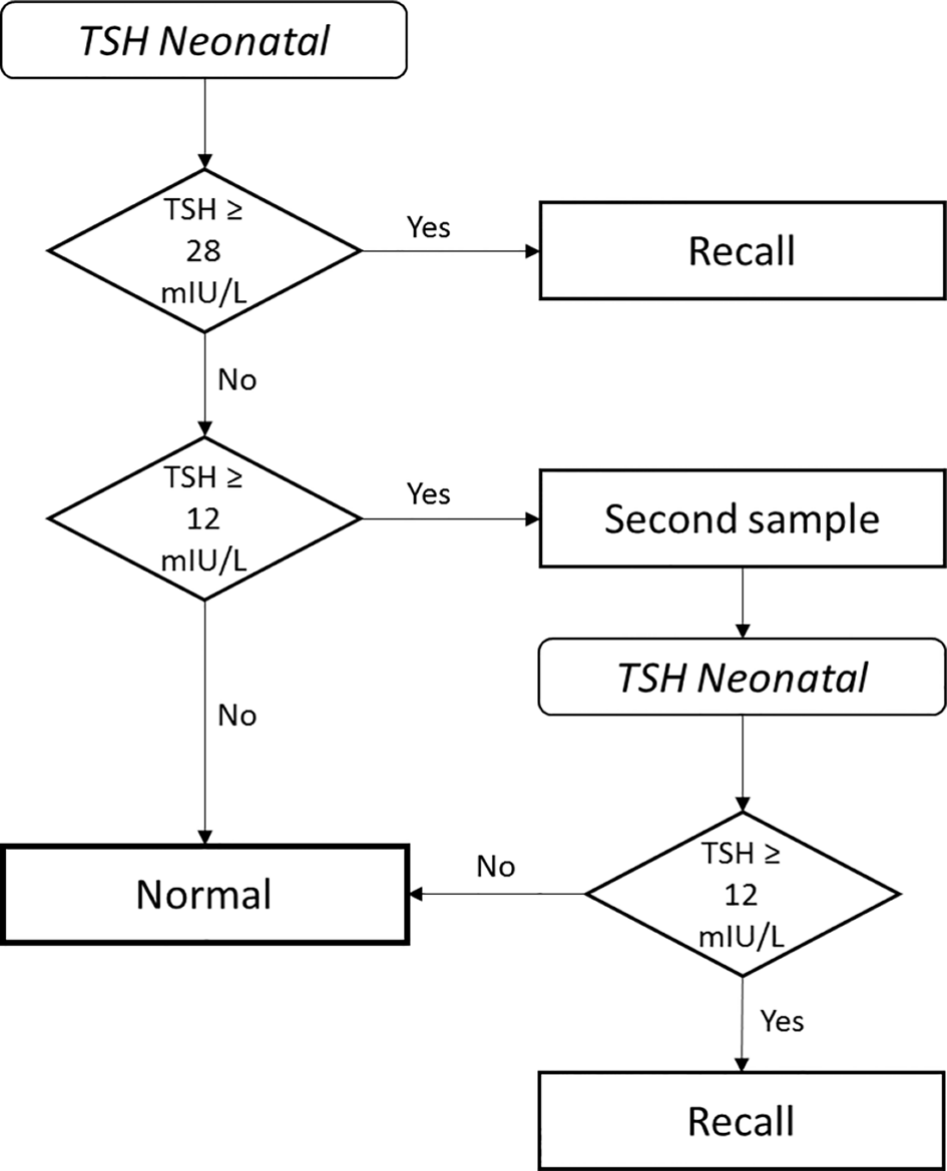
Algorithm for congenital hypothyroidism neonatal screening in Poland.

Since the 1970s neonatal screening programs for CH have been implemented worldwide (6). The blood test based on thyreotropin (TSH) concentrations determines primary PCH and TCH and transient hyperthyreotropinemia, but not secondary CH. In Poland, the program started in 1983. The scheme is presented in **Figure 1**. The threshold levels have been lowered since 2012 from TSH ≥35 mIU/ml and TSH 15-35 mIU/ml in the first and second screening, respectively. CH incidence in Poland in the first years of the neonatal screening was documented as 1:3400 – 3500. Based on the screening data, before diagnosis confirmation, the estimated incidence was 1:3888 in Poland and in the region of Warsaw 1:3185 in the years 2017-2021 (unpublished data).

The diagnosis of CH is confirmed by the thyroid function tests (TFTs) and thyroglobulin (TG) analysis and subsequent immediate treatment implementation with oral, solid or liquid, levothyroxine (L-T4). In PCH the treatment will be life-long, but up to 35% of newborns diagnosed with CH reveal to have TCH, which allows for a trial of L-T4 dose reduction or withdrawal after the critical period of neurodevelopment (17).

Timely treatment implementation with a high-dose L-T4 10-15 μg/kg/day initially, within the first two weeks of life, is recommended (1, 6, 9), although optimal L-T4 dosage is still a matter of debate. The main goal is to achieve a rapid TSH normalization and euthyreosis, which have a direct impact on a potentially reversible sequalae of CH (9). On the other hand, overtreatment periods in early childhood should also be avoided, as these may cause worse cognitive outcomes in later childhood and adolescence, although the results are not explicit (18–21).

In our study we performed the retrospective analysis of children from the neonatal period up to 3 years of age, screened, diagnosed, and treated in the Institute of Mother and Child, to determine whether there were differences between children from the first and the second screening, as well as between newborns with thyroid dysgenesis and gland *in situ* (GIS). The values of TSH, free thyroxine (fT4) serum concentrations, and L-T4 dosage at the start of the treatment and during the follow up (at 12 months and 3 years) were collected, with particular interest in the treatment strategies in regards to optimal treatment (fT4 level in the upper half of the reference range) or the therapy with initial L-T4 dose below recommended 10 μg/kg/day, and the undertreatment or overtreatment risks. The subgroups of children diagnosed based on the new, decreased TSH threshold values in the neonatal screening and children with a family history of CH were additionally analysed.

## Material and Methods

Retrospective analysis of 99 children aged 0-3 years from CH neonatal screening, treated in our clinic between 2017 and 2021, was done. There are seven laboratories involved in CH neonatal screening in Poland, with approximately 330,000-400,000 tests from 380 neonatal departments performed each year. Approximately 1/3 tests from 130 neonatal departments in five regions of Poland is analysed in the Department of Screening and Metabolic Diagnostics in the Institute of Mother and Child in Warsaw and referred to our clinic (22).

In our analysis, the inclusion criteria were referrals from CH neonatal screening. The exclusion criteria were non-CH causes of hypothyroidism. Diagnostic criteria for CH were: TSH >12.40 mIU/l and fT4 <8.37 pmol/l or normal (8.37-22.14 pmol/l); TG <0.9 ng/ml was thyroid agenesis indicator.

L-T4 treatment was implemented in all patients immediately after pediatric endocrinologist consultation, based on the same-day laboratory evaluation of the TFT and TG. All patients received treatment with the solid form of L-T4. Children had a regular follow-up, planned due to the Polish Society for Paediatric Endocrinology and Diabetology recommendations (5). L-T4 dose adjustments were made when applicable. We present the follow-up results at 12 months and 3 years of the therapy with the reference ranges for TSH 0.77 mIU/L - 7.73 mIU/l and fT4 9.44 - 17.70 pmol/l at both time points. Thyroid imaging evaluation was performed during the course of observations, with some delays due to the COVID-19 pandemic. Scintigraphy was postponed for the withdrawal trial after 3 years of life.

The results were analysed separately for the subgroup of children who completed the whole observation period until 3 years of age - complete observation group (COG) and total group of children with CH, including those younger than 3 years or not followed-up in our clinic until the age of 3 years – the general group (GG). The whole cohort was divided into the first screening group (FSG) and the second screening group (SSG), according to the screening process. Screening TSH, TFT, and TG values were examined with a chemiluminescence method – LIAISON XL, DiaSorin.

### Statistical Analysis

In statistical analysis, descriptive and inferential statistics were used. The results are presented as means ± standard deviations (SD) for normally distributed data or medians and ranges for non-normally distributed variables. The Kolmogorov-Smirnov test was used for evaluating distribution for normality. Differences between groups were assessed using Student’s t-test (or ANOVA) for normally distributed data and non-parametric Mann-Whitney test (or Kruskal-Wallis test) for non-normally distributed parameters. Chi-square test (or Fisher test) was applied to verify hypotheses regarding associations between independent categorical variables. For comparisons between different time points McNemar test was used. A p-value <0.05 was considered to be statistically significant. Statistical analysis was performed using IBM SPSS v. 25.0 software.

## Results

All patients were detected by CH neonatal screening program: 79 (79.8%) in the first screening group (FSG), and 20 (20.2%) in the second screening group (SSG). Sixty-one (61.6%) children were in the COG subgroup. Patient profiles at birth are presented in **Table 1**.

**Table 1:** Patient profiles at birth.

	Total (n = 99)	FSG (n = 79)	SSG (n = 20)	p-value
girls (n,%)	61 (61.6%)	51 (64.6%)	10 (50.0%)	0.232
TSH1, mIU/l (mean ± SD)	102.8 ± 65.6	123.9 ± 56.2	19.3 ± 4.5	<0.001
TSH2, mIU/l (mean ± SD)	23.4 ± 11.1	x	23.4 ± 11.1	x
gestational age, median (range)	39 (25-43)	39 (25-43)	39 (31-41)	0.239
Apgar score, median (range)	10 (4-10)	10 (4-10)	10 (6-10)	0.502
birth weight SDS (mean ± SD)	-0.27 ± 1.11	-0.22 ± 0.94	-0.48 ± 1.64	0.353
birth length SDS (mean ± SD)	2.10 ± 1.11	2.18 ± 1.06	1.81 ± 1.39	0.198
dysgenesis (n,%)	36 (41.9%)	33 (47.1%)	3 (18.8%)	0.038
GIS (n,%)	50 (58.1%)	37 (52.9%)	13 (81.3%)	
family history (n,%)	20 (20.2%)	13 (16.5%)	7 (35.0%)	0.065
TG <0.9 ng/ml (n,%)	6 (8.3%)	6 (10.3%)	0 (0.0%)	0.342

FSG, first screening group; SSG, second screening group; GIS, gland in situ.

### The First vs. the Second Screening Group

TSH concentrations at the start of treatment differed between FSG and SSG: ranged from 7.3 to 345 mIU/l and from 10.7 to 100 mIU/l, respectively. The median values are not provided, and the upper ranges can be biased, because part of the laboratory confirmatory tests provided only results up to the upper limit of a diagnostic method (TSH >100 mIU/l), with no further dilution analysis.

There was no association between screening TSH level and gestational age, gender, Apgar score, or birth length. However, birth weight SDS <-2 was more frequent in newborns from SSG than FSG (15.0 vs. 1.3%, p=0.014).

### Thyroid Dysgenesis vs. Gland *in situ*

In 86 (86.9% of GG) children examined with thyroid ultrasound, GIS was found in 50 (58.1%) and thyroid dysgenesis in 36 (41.9%) patients (**Table 2**). Among dysgenesis: agenesis was detected in 26 (30.3%), ectopy in two (2.3%), hemiagenesis in one (1.2%), and hypoplastic thyroid gland in seven (8.1%) cases.

**Table 2:** Ultrasonography evaluation in the examined group.

	Total (n = 86)	Dysgenesis (n = 36)	GIS (n = 50)	p-value
Girls (n,%)	51 (%)	26 (72.2%)	25 (50.0%)	0.039
TSH1, mIU/l (mean ± SD)	103.0 ± 64.9	126.0 ± 59.7	86.5 ± 64.1	0.009
TSH2, mIU/l (mean ± SD)	24.2 ± 12.3	18.7 ± 5.3	25.5 ± 13.2	0.364
Gestational age, median (range)	39 (25-42)	39 (34-42)	39 (25-41)	0.755
Apgar score, median (range)	10 (4-10)	10 (6-10)	10 (4-10)	0.015
Birth weight, g, median (range)	3400 (940-4310)	3395 (1870-4310)	3415 (940-4300)	0.707
Birth weight SDS (mean ± SD)	-0.23 ± 1.13	-0.28 ± 1.05	-0.21 ± 1.20	0.780
Birth length, cm, median (range)	54 (37-60)	55 (46-60)	54 (37-60)	0.563
Birth length SDS (mean ± SD)	2.13 ± 1.14	2.23 ± 1.21	2.05 ± 1.09	0.453
TSH1 ≥28 mIU/l (n,%)	70 (81.4%)	33 (91.7%)	37 (74.0%)	0.038
Family history (n,%)	16 (18.6%)	1 (2.8%)	15 (30.0%)	0.001
TG <0.9 ng/ml (n,%)	4 (6.6%)	3 (12.0%)	1 (2.8%)	0.296

GIS, gland in situ; TSH1, TSH in the first screening; TSH2, TSH in the second screening.

Screening TSH ≥28 mIU/l was detected in 91.7% patients with dysgenesis in comparison to 74.0% patients with GIS (p=0.038). All patients with thyroid agenesis and ectopy had screening TSH concentrations ≥28 mIU/l. In the group of patients with dysgenesis, the proportion of girls was higher than in the group with GIS (72.2 vs. 50.0%, p=0.039).

TG serum concentration was examined in 72 (72.7%) newborns; in six (8.3%) the TG level was <0.9 ng/ml (**Table 1**); in four of them the thyroid imaging was performed: agenesis was detected in three and GIS in one patient. The median TG concentration was significantly decreased in patients with dysgenesis in comparison to the patients with GIS, 85.8 ng/ml (0.0 – 2242.0) vs. 210.2 ng/ml (0.19 – 4816.0), (p=0.002), respectively. Among patients with dysgenesis, newborns with agenesis presented the lowest TG concentration.

### Treatment with Levothyroxine

We did not observe an association between TSH at the start of the treatment and birth data, gender, or thyroid dysgenesis diagnosed by ultrasonography, but there was an association with the start of the treatment day. The results of TFTs, TG, and L-T4 dose at the start of treatment, 12 months and 3 years are presented in **Table 3**. In girls fT4 at the start of the therapy was within the reference range in 36.7% and below the range in 63.3% and in boys in 63.2% and 36.8%, respectively (p=0.010). In girls the mean fT4 at the start of the treatment was 7.15 ± 5.05 pmol/l, in boys – 9.66 ± 5.36 pmol/l (p=0.022); there was no such association observed with TG, and the associations were not confirmed in further observations. We found an association between fT4 at the start of the treatment with thyroid dysgenesis – mean fT4 was significantly lower than in GIS (p=0.010). TSH levels were lower in males during the follow-up – median TSH at 12 months was 2.58 mIU/l in boys vs. 3.11 mIU/l in girls (p=0.002) and at 3 years 1.77 mIU/l vs. 2.37 (p=0.006), respectively. There was no association between the diagnostic results of the initial fT4 or TG and the start of the treatment day.

**Table 3:** Thyroid function test results and levothyroxine treatment at the start of the treatment, after 12 months and 3 years.

		Total (n = 99)	FSG (n = 79)	SSG (n = 20)	p-value
Start of the treatment (n=99)	fT4, pmol/l (mean ± SD)	8.1 ± 5.3	7.4 ± 5.2	10.9 ± 4.8	0.009
	L-T4 dose, μg/kg/d (mean ± SD)	9.3 ± 3.8	9.9 ± 3.5	6.8 ± 3.7	0.002
12 months (n=89)	TSH, mIU/l (median, range)	2.8 (0.02-82.6)	3.1 (0.02-82.6)	1.9 (0.1-13.0)	0.319
	fT4, pmol/l (mean ± SD)	16.6 ± 6.5	16.8 ± 7.1	15.8 ± 1.9	0.579
	L-T4 dose, μg/kg/d (mean ± SD)	4.0 ± 1.4	4.2 ± 1.4	3.2 ± 0.9	0.013
3 years (n=61)	TSH, mIU/l (median, range)	2.0 (0.02-20.2)	2.4 (0.02-20.2)	1.8 (0.04-9.4)	0.538
	fT4, pmol/l (mean ± SD)	18.1 ± 3.7	18.3 ± 3.7	17.3 ± 3.6	0.363
	L-T4 dose, μg/kg/d (mean ± SD)	3.3 ± 1.2	3.6 ± 1.1	2.2 ± 0.9	<0.001

FSG, first screening group; SSG, second screening group.

**Figure 2:**
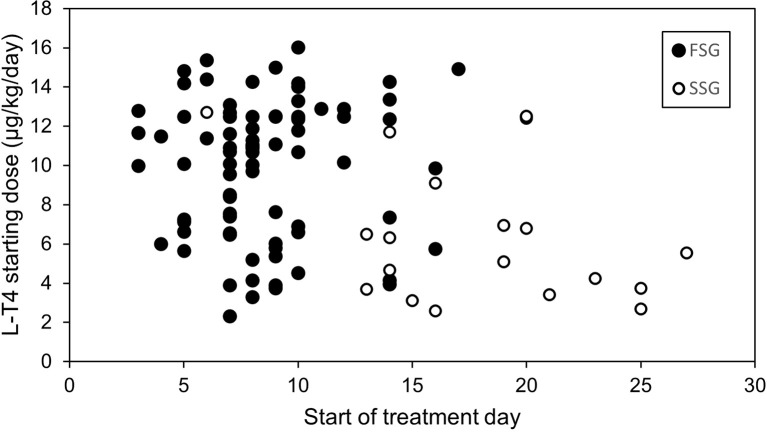
Start of the treatment day and levothyroxine starting dose in the first (FSG) and the second (SSG) screening groups.

Treatment was implemented at median of 9 days of life (3 – 27): in 8 days (3 – 17) in FSG and 19 days (6 – 27) in SSG (p<0.01) (**Figure 2**). There was no association between the start of the treatment day and TFTs at 12 months and 3 years, nor TG level. In three patients from FSG the therapy started above the recommended 14th day of life (16 - 17), and in 14 children in the SSG group (15 - 27). Eleven of these children had normal fT4 levels at the start of the treatment.

The dose of L-T4 in GG was significantly higher in the patients from FSG compared to SSG at all three analysed time points, as presented in **Table 3**. Similar observations as in GG applied to COG subgroup: mean L-T4 starting dose in FSG was 11.1 ± 3.1 μg/kg/day and in SSG – 7.7 ± 4.1 μg/kg/day (p=0.001). During the follow-up at 12 months mean L-T4 dose in FSG was 4.1 ± 1.1 μg/kg/day and in SSG – 3.2 ± 1.0 μg/kg/day (p=0.009), at 3 years of age mean L-T4 dose in FSG was 3.6 ± 1.1 μg/kg/day and in SSG – 2.2 ± 0.9 μg/kg/day (p<0.001).

We found an association at every time point in GG between L-T4 dose and thyroid dysgenesis (**Table 4**). This relationship was confirmed also in the COG: at the start of treatment – in dysgenesis mean L-T4 dose was 11.3 ± 3.2 μg/kg/day and in GIS – 9.39 ± 3.4 μg/kg/day (p=0.030), and at 3 years - in dysgenesis mean L-T4 dose was 3.7 ± 1.1 μg/kg/day and in GIS – 2.9 ± 1.3 μg/kg/day (p=0.017); such association was not observed at 12 months. We did not find an association in any of the time points between L-T4 dosage and TSH at the start of the treatment.

**Table 4:** Levothyroxine treatment and thyroid ultrasonography evaluation.

		Total (n = 86)	Dysgenesis (n = 36)	GIS (n = 50)	p-value
Start of the treatment	fT4, pmol/l (mean ± SD)	8.0 ± 5.4	6.2 ± 4.4	9.2 ± 5.8	0.010
	L-T4 dose, μg/kg/d (mean ± SD)	9.4 ± 3.7	11.0 ± 3.4	8.3 ± 3.5	0.001
12 months	TSH, mIU/l (median, range)	2.6 (0.02-82.6)	3.1 (0.03-23.9)	2.5 (0.02-82.6)	0.922
	fT4, pmol/l (mean ± SD)	16.7 ± 6.7	17.6 ± 9.2	16.1 ± 3.5	0.322
	L-T4 dose, μg/kg/d (mean ± SD)	4.0 ± 1.4	4.4 ± 1.5	3.7 ± 1.3	0.035
3 years	TSH, mIU/l (median, range)	2.0 (0.02-20.2)	1.9 (0.02-16.2)	2.3 (0.07-20.2)	0.462
	fT4, pmol/l (mean ± SD)	18.2 ± 3.7	18.6 ± 3.3	17.8 ± 4.2	0.398
	L-T4 dose, μg/kg/d (mean ± SD)	3.3 ± 1.2	3.7 ± 1.1	2.9 ± 1.3	0.017

GIS, gland in situ.

In the analysis of the therapeutic goals, we identified the group of patients with the optimal treatment according to European Society of Paediatric Endocrinology (ESPE) guidelines (2), ENDO-European Reference Network (ENDO-ERN) Consensus (1), and the Polish Society for Paediatric Endocrinology and Diabetology recommendations (9), defined as fT4 level in the upper half of the reference range during follow-up. At 12 months, 51 (58%) out of 88 patients (27 girls), and at 3 years 27 (44.3%) out of 61 patients (19 girls) were treated optimally. In this group, there were 22 (46.8%) patients with dysgenesis at 12 months and 12 (50.0%) at 3 years, compared to the rest of the group - 14 (41.2%) and 20 (58.8%), respectively. In the optimally treated group vs. the rest of the group mean L-T4 dose was 3.9 ± 1.5 μg/kg/day vs. 4.1 ± 1.3 μg/kg/day (p=0.625) at 12 months and 2.8 ± 1.1 μg/kg/day vs. 3.7 ± 1.2 μg/kg/day (p=0.005) at 3 years.

Additionally, we also analysed a group receiving L-T4 starting dose below recommended 10 μg/kg/day (2.60 - 9.86 μg/kg/day). There were 47 (47.5%) newborns in this group: 32 (40.5%) in FSG and 15 (75%) in SSG (p=0.006). At the start of the treatment mean fT4 was 9.7 ± 5.7 pmol/l compared to 6.7 ± 4.5 pmol/l in a group treated with the recommended L-T4 dose (p=0.005). The median start of the treatment day was 9 days (4 – 27) and in the rest of the cohort – 8 days (3 – 20) (p=0.038). In the follow-up at 12 months median TSH was 3.40 mIU/l vs. 1.93 mIU/l in the group receiving the recommended L-T4 starting dose (p=0.034); mean fT4 was 17.00 ± 8.78 pmol/l vs. 16.31 ± 3.63 pmol/l, respectively (p=0.163). At 3 years there were no such associations observed. In 38 patients with available ultrasound examination, GIS predominated – 29 (76.3%).

### Overtreatment and Undertreatment Risks

The analysis of overtreatment and undertreatment data is presented in **Table 5**. We divided 88 patients tested at 12 months and 61 tested at 3 years into three groups defined as (1) euthyreosis (TSH N, fT4 N) or isolated fT4 elevation (TSH N, fT4↑); (2) overtreatment: sub-hyperthyreosis (TSH↓, fT4 N) or hyperthyreosis (TSH↓, fT4↑); (3) undertreatment: sub-hypothyreosis (TSH↑, fT4 N) or hypothyreosis (TSH↑, fT4↓). We included patients with the isolated fT4 elevation (eight patients, 9.1% at 12 months and 16, 26.2% at 3 years) to the euthyreosis group, as it can be attributed to compliance issues.

**Table 5:** Overtreatment and undertreatment in follow-up after 12 months and 3 years.

		Total (n = 99)	FSG (n = 79)	SSG (n = 20)	p-value
12 months (n,%) n=88	Euthyreosis	54 (61.4%)	42 (59.2%)	12 (70.6%)	0.799
	Overtreatment	19 (21.6%)	16 (22.5%)	3 (17.6%)	
	Undertreatment	15 (17.0%)	13 (18.3%)	2 (11.8%)	
3 years (n,%) n=61	Euthyreosis	39 (63.9%)	30 (62.5%)	9 (69.2%)	1.000
	Overtreatment	15 (24.6%)	12 (25.0%)	3 (23.1%)	
	Undertreatment	7 (11.5%)	6 (12.5%)	1 (7.7%)	

FSG, first screening group; SSG, second screening group.

The euthyreosis was observed in 54 patients (61.4%) at 12 months and in 39 patients (63.9%) at 3 years of the follow-up, and there was no association between treatment status and FSG and SSG groups. At both time points the group of overtreated predominated over the undertreated patients: at 12 months 19 (21.6%) vs. 15 (17%), at 3 years 15 (24.6%) and seven (11.5%), respectively. At 12 months there was also a substantial group with hypothyreosis, which diminished at 3 years time point. In the overtreatment group, mean starting L-T4 dose was 10.7 ± 2.6 μg/kg/day, 4.2 ± 0.8 μg/kg/day after 12 months, and 3.6 ± 1.1 μg/kg/day at 3 years. We did not find an association between the treatment status and FSG/SSG, the start of the treatment day, gender, or GIS in ultrasonography.

**Figure 3:**
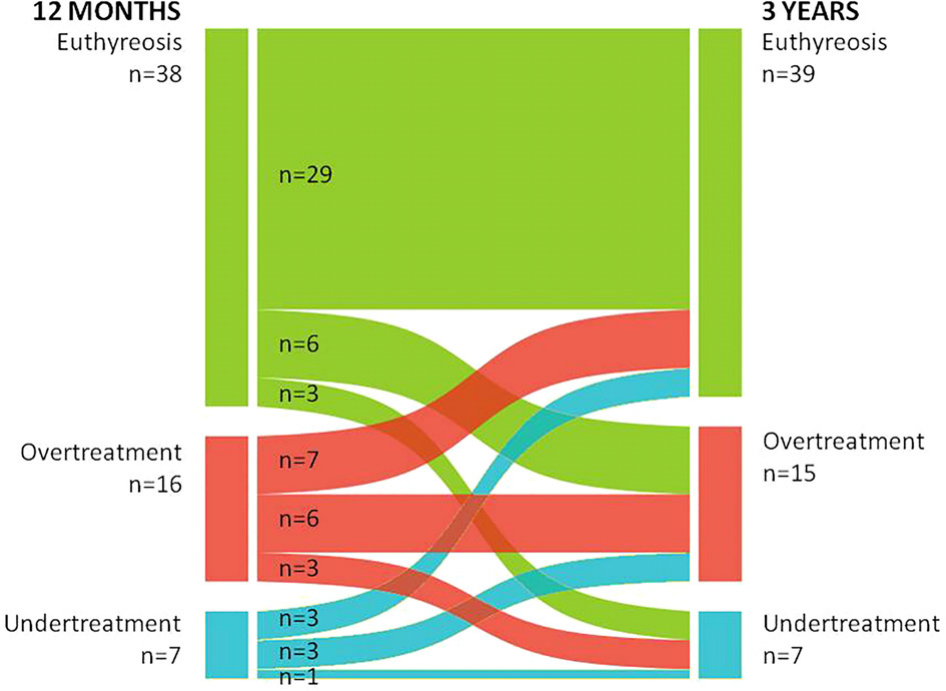
Comparison of the treatment status at 12 months and 3 years.

Additionally, we compared patients’ treatment status between time points 12 months and 3 years. Data are presented in **Figure 3**. We observed that among patients followed up for 3 years (COG) the proportions of euthyreosis were similar at both time points – 38 (62.3%) vs. 39 (63.9%), the hyperthyroidism group also remained similar – 16 (26.2%) vs. 15 (24.6%), and seven (11.5%) patients had hypothyroidism at both time points. Seven patients did not change treatment status – six remained in overtreatment and one in undertreatment group.

### New TSH Threshold Values

We analysed the data of two subgroups of patients, who were qualified for CH based on the new screening threshold range, recommended in Poland since 2012, with screening TSH values of 12 - 15 mIU/l (n=3) and 28 - 35 mIU/l (n=7). The results are presented in **Table 6**.

**Table 6:** Initial characteristics, thyroid function tests, and levothyroxine dosage in subgroups in relation to the previous TSH threshold values.

		12-15mIU/l (n = 3)	15-28mIU/l (n = 17)	28-35mIU/l (n = 7)	≥35mIU/l (n = 72)	p-value
Dysgenesis (n,%)		1 (33.3%)	2 (15.4%)	2 (28.6%)	31 (49.2%)	0.121
GIS (n,%)		2 (66.7%)	11 (84.6%)	5 (71.4%)	32 (50.8%)	
Family history (n,%)		1 (33.3%)	6 (35.3%)	3 (42.9%)	10 (13.9%)	0.081
TG <0.9 ng/mL (n,%)		0 (0.0%)	0 (0.0%)	0 (0.0%)	6 (11.5%)	0.508
Start of the treatment (n = 99)	fT4, pmol/l (mean ± SD)	12.4 ± 2.5	10.6 ± 5.2	14.6 ± 1.6	6.7 ± 4.9	<0.001
	L-T4 dose, μg/kg/d (mean ± SD)	3.9 ± 1.0	7.3 ± 3.7	6.7 ± 3.3	10.2 ± 3.4	<0.001
12 months (n = 89)	TSH, mIU/l median (range)	2.8 (1.6-3.4)	3.6 (0.1-13.0)	2.8 (0.02-16.0)	3.4 (0.02-82.6)	0.796
	fT4, pmol/l (mean ± SD)	14.8 ± 3.6	16.0 ± 1.4	16.8 ± 2.3	16.8 ± 7.4	0.940
	L-T4 dose, μg/kg/d (mean ± SD)	2.5 ± 0.7	3.4 ± 0.9	2.9 ± 1.1	4.3 ± 1.4	0.003
3 years (n = 61)	TSH, mIU/l median (range)	1.4 (1.0-1.8)	2.0 (0.04-9.4)	1.3 (0.5-20.2)	2.6 (0.02-16.2)	0.872
	fT4, pmol/l (mean ± SD)	14.5 ± 3.1	17.8 ± 3.5	17.2 ± 3.2	18.5 ± 3.8	0.450
	L-T4 dose, μg/kg/d (mean ± SD)	1.3 ± 1.8	2.4 ± 0.6	3.1 ± 1.1	3.7 ± 1.1	0.001

We did not find differences between these groups and the rest of the cohort regarding the birth characteristics. Interestingly, three patients (42.9%) from the group with TSH 28 - 35 mIU/l had a family history of CH, compared to 10 patients (13.9%) with screening TSH ≥35 mIU/l, but also in the group with TSH 12 - 15 mIU/l there was one child with a positive family history. The frequency of dysgenesis was comparable in both groups, one and two patients, respectively.

All the children in the group with TSH 12 - 15 mIU/l had a good treatment outcome in terms of TFTs results during the follow-up. This effect was achieved with relatively lower L-T4 doses. Similarly, in the group with TSH 28 - 35 mIU/l, most children had TSH within the normal reference range during the follow-up. However, more patients showed higher fT4 values.

### Family History of Congenital Hypothyroidism

In our cohort, 20 children (20.2%), including 11 girls (55%), had a family history of CH; having an older sibling with CH. Interestingly, they were present in both FSG (n=13, 16.5% of GG) and SSG (n=7, 35% of GG), but with a tendency toward a higher risk in the SSG (p=0.065). Among the patients with available ultrasonography evaluation, only one patient had thyroid agenesis, and 15 children presented GIS. There were no differences in the TFTs during the follow-up, except surprisingly higher fT4 value at the start of the therapy compared to the patients with no CH family history (11.18 ± 6.11 vs. 7.34 ± 4.80 pmol/l, p=0.003). L-T4 dose was lower at the start of the treatment and at 3 years of follow-up (6.91 ± 3.56 vs. 9.86 ± 3.58, p=0.001 and 2.18 ± 1.54 vs. 3.44 ± 1.12 μg/kg/day, p=0.014, respectively). This difference was also shown for six patients with the positive family history in COG.

## Discussion

The pathogenesis of CH is complex and it is well known that it can be influenced by environmental and genetic factors. The genetic background was not examined in our study. The risk factors for neonatal CH, transient, or permanent vary between countries and include gender, premature birth, worse newborn health status, and children born as small for gestational age (SGA) (23–26). In our cohort mean fT4 level at the start of treatment was lower in girls, but despite it, no significant difference was observed in the initial L-T4 dose. Among newborns with thyroid dysgenesis, females were predominant (72%), and that was also observed by the other authors (27, 28).

Thyroid dysgenesis remains the most common cause of CH, although dyshormonogenesis frequency seems to be rising (6, 14). However, in our study among children examined with thyroid ultrasound, GIS was found in most cases (58.1%), but the newborns with dysgenesis (both agenesis and ectopy) predominated in FSG. As expected, we found lower mean fT4 at the start of the treatment in patients with thyroid dysgenesis compared to GIS. It was reflected also in the initial L-T4 dosage which was higher in children with dysgenesis, which allows predicting the increased risk of the life-long need for the treatment in these patients (29).

It is still discussed whether the screening TSH can predict the transient or permanent forms of CH. The important question is if the screening TSH test can be useful to select the patients who could be treated less extensively to avoid overtreatment and reduce parents’ anxiety and medical care costs (30–32). It is known from other data that screening TSH tends to be lower in neonates with TCH than in those with PCH (17, 33–35). In our study, decreased screening TSH results were observed in newborns with birth weight below -2 SDS who are mostly burdened with TCH risk. Therefore early (3 up to 5 days of life) determination of TSH and fT4 concentration in the serum in these groups of children, regardless of the CH screening program, is recommended in Poland (9, 36).

Di Dalmazi et al. showed that neonatal TSH can be influenced by gender: male newborns had higher TSH concentrations than females, and by gestational age: preterm newborns had lower TSH values than the term ones (34, 37). In our study, we also observed a tendency toward lower screening TSH in preterm newborns. In contrast, according to Bosh-Gimenez et al., TSH concentrations were found to be higher in neonates born with SGA (38).

The results of TG values in the studied cohort are not explicit and there is a need for further evaluation of this particular examination. TG is essential for TH synthesis and homozygous or compound heterozygous mutations of the TG gene can result in PCH (39). Most often TG concentration is increased in patients with dyshormonogenesis. Although most patients with CH due to TG gene mutations show decreased serum TG levels (40), in some rare cases elevated serum TG was observed (39). In our study, median TG was decreased in newborns with dysgenesis in comparison to GIS, as expected the lowest TG concentrations were detected in newborns with agenesis. In our opinion, patients with positive results for TG and absent thyroid gland in ultrasonography need to be the first-line group for the thyroid scintigraphy, due to the possibility of the ectopy. However, also in the patients with CH and GIS, with possible dyshormonogenesis, the scintigraphy can add additional diagnostic value, as these patients may have an iodine uptake/organification defect (12). In our study, the goiter was not observed in the examined group, but according to the recent concept, the patients with CH and GIS may present some form of dyshormonogenesis despite not presenting the classical goitrous form of CH (14).

According to the recommendations, treatment for CH should be started as soon as possible, within 14 days of life, with no delay for diagnostic procedures (1, 6, 9). In our study, the treatment predictably started earlier and with higher L-T4 doses in FSG than in SSG. In patients with delayed treatment implementation, the reason was mainly due to extended screening procedures (SSG) and technical problems with the patients’ arrival for endocrine evaluation. However, most of these patients had normal fT4 levels at the start of the treatment.

The goal of the treatment is to obtain and maintain fT4 levels within the upper half of the reference range (1, 6, 9). In our study, this group consisted of 58.0% and 44.3% of children in subsequent time points, and we did not find any association with other analysed factors like birth data, gender, or ultrasonography evaluation. We suspect that the most important factors to obtain the optimal treatment requirement are compliance in terms of method of L-T4 administration, daily adherence, meal time distance, avoiding foods that influence L-T4 absorption (i.e., soy formula, calcium or iron supplements), and families understanding of the critical importance of the regular therapy (9, 29). Also isolated fT4 elevation may be attributed to compliance issues related to taking the L-T4 dose shorter than 4 hours before blood sampling or chronic poor adherence with “making up” by taking multiple doses before the scheduled test and endocrine consultation (29). However, also with optimal compliance, some patients seem to require higher L-T4 doses to normalize TSH values, which may suggest a form of “T4 resistance” (21, 29).

At the 12 months time point the mean L-T4 dose in the SSG was 3.2 μg/kg/day, which according to ENDO-ERN consensus, could suggest TCH in patients with GIS and is close to the rationale for the need for the treatment re-evaluation after 6 months of age (recommended L-T4 dose <3 μg/kg/day) (1). Also, the differences in L-T4 dosages between FSG and SSG groups in both follow-up time points supported the above data. Although the median L-T4 doses in both groups at 12 months and 3 years were lower than established in Polish recommendations (3) the disease control in terms of TFTs results showed a higher risk for over- than undertreatment. The undertreatment was present in 17% of the study group at 12 months and 11.5% at 3 years. Only one patient remained undertreated both at 12 months and 3 years. In other analysed studies undertreatment prevalence was 12/61 (19.6%) (19), 24/55 (46%) (20), and 11/88 (13%) (21).

According to a German group, episodes of undertreatment in the first 3 months of life led to increased scores in the Withdrawn, Anxious, Social, and Thought (WAST) test used for an autism diagnosis. Also, children with severe CH undertreated at 3-6 months of age were more likely to have anxiety and introversion social problems compared to the rest of the group (20).

In respect to decreasing threshold values in neonatal screening and subsequently including in the treatment the newborns with mild CH, mostly with GIS confirmed in ultrasonography evaluation, the question arises if high-dose L-T4 treatment strategy should not be reserved for the patients with severe primary CH (3–5, 14). In our study, we assessed separately the group treated with the starting L-T4 dose of <10 μg/kg/day. It was a relatively large group of 47 children (47.5%) with mean fT4 within the reference range, and 76.3% of these patients with available ultrasonography examination had GIS, which can be attributable to TCH. The rate of TCH is established as 7% – 35% in different studies (10, 17). Analysis of other TCH predictors in German and Austrian databases, apart from GIS, were TSH at the start of the treatment <73 mIU/l and median L-T4 dosage 3.1 μg/kg/day at 12 months and 2.9 μg/kg/day at 2 years (17).

There is an extensive discussion regarding proper L-T4 dosing in respect to the long-term neurocognitive outcome in children with CH, how to keep the beneficial effect of prompt and effective treatment, and to avoid risks of side effects at the same time. Currently recommended starting L-T4 dose often (up to 60% of patients) leads to overtreatment (8, 16, 29). In our study, the overall overtreatment rate was 21.6% after 12 months and 24.6% after 3 years, with L-T4 doses not exceeding the recommended range. Frequent follow-up at the beginning of the treatment warrants the proper dose adjustments. Moreover, ESPE and ENDO-ERN recommendations state that, in case of obtaining TFTs with elevated fT4, dose reduction should not be based on a single result, unless evident TSH suppression (1, 6).

The overtreatment episodes may have clinical implications, although the data are inconsistent. A Spanish study, where 50 children with CH were observed, revealed a relation of the number of overtreatment episodes in the first 6 months with alertness deficits at school age – inability to maintain focus of attention or respond appropriately and resist inappropriate behaviours (18). A German study based on an analysis of 61 children with CH up to 11 years suggested that overtreatment during the first 2 years had a subtle negative impact on cognitive development, compared to undertreatment (19). In another study of the above group, with an evaluation of 55 patients, the association of the Attention, Delinquency, and Aggression (ADA) score (used in the diagnosis of attention deficit hyperactivity syndrome, ADHD) with overtreatment in 1 – 3 months of life was reported (16). However, in the meta-analysis performed by Aleksander et al., including 438 patients, the authors pointed out that the recommended L-T4 dosage is safe and efficient, and lower doses can lead to 6-8 points lower IQ in children with severe CH (21). In our study, the group with overtreatment was more frequent than undertreatment at both time points of the follow-up. In this group, six patients who were overtreated at 12 months, were also overtreated at 3 years of age.

The last decade brought a wide discussion regarding the notable increase in the incidence of CH in regard to changing the TSH thresholds criteria in many screening programs throughout the world (3–5, 14, 35, 40). It has been observed in many studies that the lowering of TSH screening values has enabled clinicians to identify more children with suspected CH. However, part of this group most probably represents either mild or TCH. It has been discussed whether the treatment of these patients is indeed necessary.

In our cohort, we observed similar results, after lowering the TSH threshold values in the two subgroups of patients qualified for treatment based on revised, lowered screening criteria. Mainly mild hypothyroidism with the mean fT4 values within the reference range and GIS was present. Moreover, after the 12 months and 3 years of L-T4 treatment, the TFTs showed either euthyroid state or overtreatment while treated with lower L-T4 doses in comparison to the rest of the cohort. Most authors recommend treating the patients with the milder form of the disease after fulfilling the changed screening criteria, at least in the first years of life. This approach seems to be appropriate even if the therapy influence on the neurocognitive function in these milder cases, especially with normal fT4 results at the start of the treatment, has not been fully evaluated yet (3, 4, 35). In borderline cases of mild hypothyroidism, there is a possibility to consider the LT4 treatment withdrawal after 12 months (6, 41). However, the Polish Society for Paediatric Endocrinology and Diabetology recommends performing the re-diagnostic process after the 3 years of life (9). This re-evaluation is planned in our study group after 3 years of life, together with the thyroid scintigraphy examination.

Regarding CH family history, surprisingly we found only one case of thyroid dysgenesis in this group. Moreover, fT4 at the start of the treatment was higher in this group compared to the rest of the cohort. This again may implicate a wide pathogenesis background of CH, with the possible dyshormonogenesis background associated with milder CH. Therefore, genetic studies, especially in families with more than one child affected, are needed.

Our study has its limitations, mainly limited imaging diagnostic examinations, without the thyroid scintigraphy, that could have explained in more detail the pathogenesis of CH in the presented children. We were not able to present the genetic background of the patients as well. Moreover, a lack of precise actual TSH levels following dilution at the diagnosis is a significant limitation of the initial data analysis. The strengths of the research consist of a relatively large group of patients followed up in a single centre with one diagnostic and therapeutic approach to the children with CH. Three points of control were selected for the analysis to decrease the vast amount of data. Further evaluation of more detailed observation, especially in between the visits during the first year of life, is planned.

## Conclusions

The analysis of the 5-year experience in the single centre confirms the general efficacy of the CH diagnostic pathway and the timely implemented L-T4 therapy. The imaging examinations, thyroid ultrasound, or even more sufficient thyroid scintigraphy, seem to have a significant role in the diagnostic and therapeutic process in CH, as higher L-T4 doses could be recommended to patients with thyroid dysgenesis.

However, the results show a relatively significant risk for overtreatment, presented in 1/5 of the study group after 12 months and 1/4 after 3 years of the L-T4 therapy. The suspected overtreatment after the first 12 months of L-T4 therapy requires more detailed observation with consideration of the diagnosis re-evaluation process. Together with a recent more frequent diagnosis of mild hypothyroidism in newborns, clinicians may consider an earlier trial of lowering the L-T4 dose and possible withdrawal of the treatment. The recommended frequency of clinical appointments should be respected to detect and prevent the over- and undertreatment episodes in children with CH during the follow-up.

## Data Availability Statement

The raw data supporting the conclusions of this article will be made available by the authors, without undue reservation.

## Author Contributions

All authors have accepted responsibility for the content of the manuscript and approved submission. EL contributed to the plan of the study, prepared basic materials, took part in data collection, wrote and revised the manuscript. AL-A contributed to the plan of the study, wrote and revised the manuscript. DW contributed to data collection, wrote and revised the manuscript. KS, EM performed the statistical analysis of the study and revised the manuscript. MO contributed to up-to-date data of the neonatal screening program.

## Conflict of Interest

The authors declare that the research was conducted in the absence of any commercial or financial relationships that could be construed as a potential conflict of interest.

## Publisher’s Note

All claims expressed in this article are solely those of the authors and do not necessarily represent those of their affiliated organizations, or those of the publisher, the editors and the reviewers. Any product that may be evaluated in this article, or claim that may be made by its manufacturer, is not guaranteed or endorsed by the publisher.
